# Efficacy comparison of antibiotic bone cement–coated implants and external fixations for treating infected bone defects

**DOI:** 10.1007/s00264-023-05727-8

**Published:** 2023-03-02

**Authors:** Linhu Wang, Shuaikun Lu, Wen Luo, Guoliang Wang, Zhenfeng Zhu, Yunyan Liu, Hao Gao, Congxiao Fu, Jun Ren, Yunfei Zhang, Yong Zhang

**Affiliations:** 1 Department of Orthopaedics, Second Affiliated Hospital, Air Force Medical University, 1 Xinsi Rd, Xi’an, 710038 Shaanxi China; 2 Department of Ultrasound, Xijing Hospital, Air Force Medical University, 169 Changlexi Rd, Xi’an, 710032 Shaanxi China

**Keywords:** Anti-bacterial agents, Bone cements, Bone diseases, infectious, Fracture fixation, Retrospective studies

## Abstract

**Purpose:**

This study aimed to investigate the clinical efficacy of antibiotic bone cement–coated implants compared with external fixations for treating infected bone defects.

**Methods:**

We retrospectively enrolled 119 patients with infected bone defects in our hospital from January 2010 to June 2021, of which 56 were treated with antibiotic bone cement–coated implants and 63 were with external fixation.

**Results:**

The pre-operative and post-operative haematological indexes were tested to assess the infection control; the post-operative CRP level in the internal fixation group was lower than that in the external fixation group. No statistical significance was found in the rate of infection recurrence, loosening and rupture of the fixation, and amputation between the two groups. Twelve patients in the external fixation group had pin tract infection. In the evaluation of the Paley score scale, bone healing aspect revealed no significant difference between the two groups, while in the limb function aspect, antibiotic cement–coated implant group showed a much better score than the external fixation group (*P* = 0.002). The anxiety evaluation scale result also showed lower score in the antibiotic cement implant group (*P* < 0.001).

**Conclusions:**

Compared with external fixation, antibiotic bone cement–coated implant had the same effect on controlling infection and was more effective in recovering limb function and mental health in the first-stage treatment of infected bone defects after debridement.

## Introduction

Infectious bone defects are mostly caused by open fractures with a serious contamination, infection after internal fixation of fractures, and haematogenous osteomyelitis. The treatment involves both infection control and repair of bone defects [1, 2]. The conventional treatment strategy is mainly completed through a two-step procedure. The first stage of treatment is to thoroughly debride the infected or necrotic tissue and apply antibiotics locally/systemically to control infection and stabilize the bone defect site by fixation devices. The second stage of surgery is mainly to select the appropriate material to repair the bone defect.

It is a challenge for clinicians to repair bone defects in the presence of infection. Whether the infection can be eliminated or not has become the premise and key to the healing of infected bone defects. On the one hand, local acidic metabolites caused by infection also enhance osteoclastogenesis and induce osteoblast apoptosis, hindering the repair of bone defects [3]. On the other hand, bacteria compete with the host tissue to settle on the surface of the implant. Once the bacteria adhere to the surface of the implant, they form microcolonies and biofilms that protect them from the immune system by encasing them in an abundant matrix composed of extracellular polymeric substances (EPSs) [4]. This allows them to persist in hostile host environments, while the acidic environment of infection accelerates this process [5], leading to recurrent infections and even amputation, resulting in a heavy economic and psychological burden on patients. Some researchers suggest that treatment should follow the following principles to achieve better infection control: (1) thorough debridement to remove infection and necrotic tissue (2) as much as possible to maintain the stable structure of the bone defect (3) to ensure localized soft tissue coverage and (4) local and systemic antibiotic therapy [6]. Researchers have found that the stabilization of the bone defect helps control infection after thorough wound debridement [7, 8]. But the use of internal fixation in the first stage after debridement may enhance the risk of bacterial infection of the implant surface due to bacterial colonization. Therefore, the traditional treatment method is to use an external fixator [9, 10]. Although it reduces the risk of infection from internal metal fixation, it also has many disadvantages: poor stability, especially for large segments; high incidence of pin tract infection; long-term wearing having negative effects on the patient’s mental health; and limited daily physical activity of patients, which is not conducive to early functional exercise [11–14].

In addition, the avascular nature of the sequestrum prevents systemic antibiotics from reaching the treatment site. Also, the local concentration of intramuscular and intravenous antibiotics in infected bone tissue is low due to the defect of a large amount of bone tissue at the lesion site in patients with infected bone defects. The concentration is not enough to kill bacteria or biofilms [15, 16], and long-term intravenous antibiotics may cause dysbiosis, liver and kidney damage, and drug resistance in the body. With the continuous advance of bone cement technology, some researchers began to apply antibiotic bone cement–covered internal fixation to the treatment of infected bone defects [17–19], achieving good results. Locally placed antibiotic bone cement not only avoids the side effects of high-dose systemic intravenous antibiotics but also forms high-concentration antibiotics locally, persistently killing bacteria. Case–control and prospective studies exploring the effect of antibiotic bone cement–coated implant are few. This study aimed to compare and analyze the clinical efficacy of antibiotic bone cement–coated implants and external fixation after primary debridement in patients with infected bone defects to provide a decision-making basis in clinical practice.

## Materials and methods

This retrospective analysis was performed on patients with infected bone defects admitted to the Second Affiliated Hospital of Air Force Medical University from January 2010 to June 2021. It was approved by the ethics committee of the hospital (No. K202204-14). We fully respected the privacy of patients and obtained informed consent from all patients prior to surgery. The diagnosis of infected bone defects was mainly on the basis of history, signs, and symptoms including localized pain and swelling, imaging studies (X-ray, computed tomography, radionuclide bone scan, and magnetic resonance imaging) showing bone destruction or changes, microbiology, histopathology, and laboratory tests. The diagnosis was made after comprehensive consideration by clinicians, and the histopathological examination was the gold standard.

### Inclusion criteria

1. Patients diagnosed with an infected bone defect in the hospital
2. One-stage application of antibiotic bone cement–coated implants or external fixators after debridement
3. Follow-up time ≥ 12 months

### Exclusion criteria

1. Patients with malignant tumors, immunocompromised or acquired immunodeficiency syndrome, diabetes, or severe malnutrition
2. Affected limb having serious vascular and nerve damage with no limb salvage value
3. Patients with incomplete clinical and follow-up data

### Treatment method (patients in two groups were underwent a staged operation procedure)

#### First-stage treatment

##### Thorough debridement

The areas of bone and soft tissue necrosis were assessed on imaging prior to surgery, and debridement was performed until bleeding and viable bone at the resection margin (characterized by the “paprika sign”). The necrotic tissue, sequestrum, and old fixation were completely removed during debridement, and the deep tissue was collected for bacterial culture and pathological examination. After debridement, a large amount of hydrogen peroxide and normal saline were used to irrigate the wound repeatedly. Then, iodophor was used to disinfect the surgical area again, the surgical instruments were replaced, the operating sheet was relaid, and the extent of the bone defect was recorded.

##### Stabilization of the bone defect

**Internal fixation group**

**Fig. 1:**
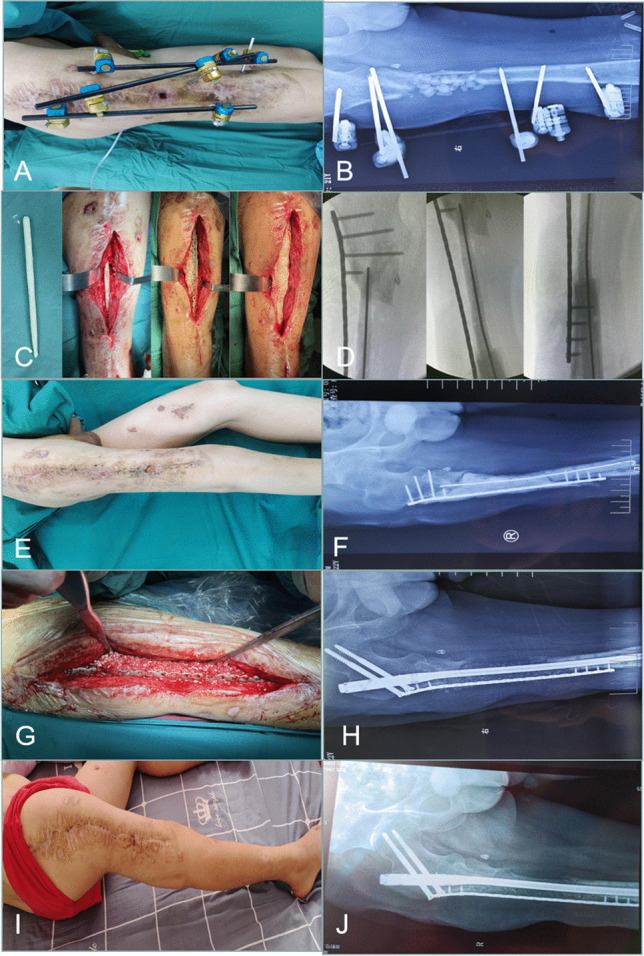
**A** infected limb with sinus and external fixator before the surgery. **B** X-ray of infected femoral bone before the surgery. **C** After a thorough debridement, antibiotic bone cement–coated nail and plate were used to stabilize the bone fracture, and the bone defect was filled with antibiotic cement spacer. From left to right: bone cement–coated K-wire was placed in the femoral medullary cavity. The steel plate was attached to the outside for reinforcement. **D** X-ray of the mid and lower level of the femoral cavity during the surgery. **E**, **F** Pictures after stage I surgery. **G**, **H** Autologous iliac bone graft combined with calcium sulfate and calcium phosphate composite materials filled into the bone defect cavity. **I**, **J** Post-operative X-ray and the incision healed at the 3-month follow-up

Plates or intramedullary nails were used to fix the defect end according to different bone defect sites and intramedullary infection. The broken end of the defect was filled with polymethyl methacrylate (PMMA) bone cement containing gentamicin. Subsequently, 2 g of antibiotic (vancomycin or imipenem) powder was added to 40 g cement powder, fully crushed, stirred evenly, and then mixed well with the liquid monomer. When the mixed antibiotic bone cement was in the dough stage, the bone defect was filled, the internal fixation plate or intramedullary nail was wrapped and extended 5–6 cm to the proximal and distal ends of the bone defect, and the bone cement was smoothed. When the bone cement solidified and heated up, ice-cold saline was used for flushing to reduce thermal damage to surrounding tissues. A typical case is shown in Fig. 1.

**External fixation group**

With the help of intraoperative fluoroscopy, external fixation instruments were used to stabilize the bone defect. The proximal and distal fixation pins were kept away from the infected area, and the important blood vessels and nerves were avoided during needle insertion. The broken end of the defect was also filled with the mixed antibiotic bone cement.

#### Second-stage treatment

The second stage of surgery was mainly to repair the bone defect. As the infection was under control, antibiotic bone cement was removed, and the defect was repaired with autologous or allogeneic bone grafting. Afterwards, either internal fixation or external fixation was used for skeletal stabilization in two groups.

### Post-operative treatment and follow-up

Broad-spectrum antibiotics were used at the beginning of treatment. Sensitive antibiotics were applied instead after the drug susceptibility and bacterial culture results were obtained. Intravenous and oral antibiotics were given for about four to six weeks. After the wounds healed, the patients in both groups could perform functional exercises of the affected limbs and early local weight-bearing training under the protection of braces. The laboratory examination, including white blood cell (WBC) counts, erythrocyte sedimentation rate (ESR), and C-reactive protein (CRP) levels, was conducted regularly after the surgery, and whether the infection was under control was evaluated six months later. The infection control criteria were as follows. Significant cure was defined as no signs or symptoms of bone infection for at least six months following treatment. Recurrence was defined as infection occurring at the same site requiring debridement treatment or systemic and local application of antibiotics. The imaging studies were performed to evaluate the effect of therapy one month, three months, six months, nine months, and 12 months after the surgery.

### Data collection and evaluation

The patients were followed up every three months after the surgery to obtain baseline data such as age, sex, aetiology (traumatic/haematogenous), smoking history, Cierny–Mader classification (anatomic type), Cierny–Mader classification (physiological type), sinus tract positive rate, bacterial culture positive rate, length of bone defect, bone infection time, and disease site.

### Infection control

Pre-operative and post-operative local signs or symptoms, laboratory tests, bacterial culture results, histopathology for the analysis of infection control, and imaging data including X-rays and computed tomography scans of the affected limb were included. The final results were evaluated by two independent surgeons.

The criteria for bone union were as follows. The clinical judgment of fracture healing usually relied on physical examination, including the absence of pain at the fracture site when the affected limb was weight bearing, no local tenderness, percussion pain, and weight-bearing capacity of the affected limb. The radiographic evaluation was based primarily on radiographs and typically included a minimum of three cortices bridging on two perpendicular X-rays of the defect site and loss of fracture line shadow and cortical continuity [20]. Limb function and bone healing were assessed according to Paley’s criteria [21]. The Self-rating Anxiety Scale (SAS) was used to assess the mental health of patients. The result was divided into mild, moderate, and severe anxiety according to the total score.

### Statistical analysis

SPSS 26.0 statistical software was used to process data. The continuous variables were expressed as “mean ± standard deviation” or median and interquartile range (IQR), and the dichotomous variables were expressed as the number of cases or percentages. The dichotomous variables were compared using the chi-square test or Fisher’s exact test. Student’s *t*-test was used to compare the means and the Mann–Whitney test was used to compare non-parametric data in the two groups, with *P* < 0.05 indicating a statistically significant difference.

## Results

### Patient characteristics

A total of 119 patients (56 in the internal fixation group and 63 in the external fixation group) participated in the study. The patients in both groups were classified according to age, sex, aetiology (traumatic/haematogenous), smoking history, Cierny–Mader classification (anatomic type), Cierny–Mader classification (physiological type), sinus tract positive rate, bacterial culture positive rate, length of bone defect, and bone infection time, with no statistically significant difference between the groups (Table 1).

**Table 1:** Clinical characteristics of patients

Parameters	Internal fixation (*n* = 56)	External fixation (*n* = 63)	*P* value
Sex			
Male	44	49	0.917
Female	12	14	
Age (year)	47.57 ± 14.44	43.16 ± 13.34	0.086
Smoker			0.571
Yes	26	26	
No	30	37	
Bone defect (cm)	6.99 (4.19 ~ 11.13)	6.00 (4.00 ~ 9.30)	0.109
Duration of infection (month)	4.00 (1.25 ~ 12.00)	2.00 (1.00 ~ 7.00)	0.075
Cierny–Mader classification^1^			0.190
A	15	24	
B	41	39	
Cierny–Mader classification^2^			0.850
III	16	19	
IV	40	44	
Etiology			0.892
Hematogenous	4	6	
Posttraumatic	52	57	
Previous implant			0.504
Nail	6	12	
Plating	16	13	
External fixator	6	5	
None	28	33	
Patients with sinus	32	45	0.104
Bacterial culture results			0.104
Positive	36	49	
Negative	20	14	
The amount of debridement	1.32 ± 0.74	1.47 ± 0.78	0.190

^1^Physiological type; ^2^Anatomic type

### Primary outcomes

No statistically significant difference was found in the recurrence rate of infection, the loosening and rupture of the fixator, and amputation rates between groups, but 12 patients with external fixation had pin tract infection (Table 2). The results of the Paley score on bone healing indicated no statistically significant difference between the two groups. Still, the results in the internal fixation group were better than those in the external fixation group in terms of limb function. The number of patients with mild, moderate, and severe anxiety was 35, 17, and four in the internal fixation group, respectively, and 18, 35, and ten in the external fixation group, respectively (Table 3). The anxiety level was lower in patients with internal fixation than in those with external fixation.

**Table 2:** Related complications during the follow-up

Parameters	Internal fixation (*n* = 56)	External fixation (*n* = 63)	*P* value
Infection recurrence*	4	8	0.315
Pin tract infection	0	12	< 0.001
Fixator loose shift or break	2	2	1.000
Amputation	3	9	0.106

^*^The infection recurrence rate here refers to the infection after the first debridement, and the nail canal infection in the external fixation group is not included as recurrence

**Table 3:** Paley and SAS assessment of patients

Parameters	Internal fixation (*n* = 56)	External fixation (*n* = 63)	*P* value
Paley’s score			
Evaluation of bone healing			0.083
Excellent	29	20	
Good	13	24	
Fair	2	3	
Poor	12	16	
Joint function evaluation			0.002
Excellent	28	15	
Good	15	18	
Fair	9	22	
Poor	4	8	
SAS assessment			< 0.001
Mild anxiety	35	18	
Moderate anxiety	17	35	
Severe anxiety	4	10	

### Secondary outcomes

**Fig. 2:**
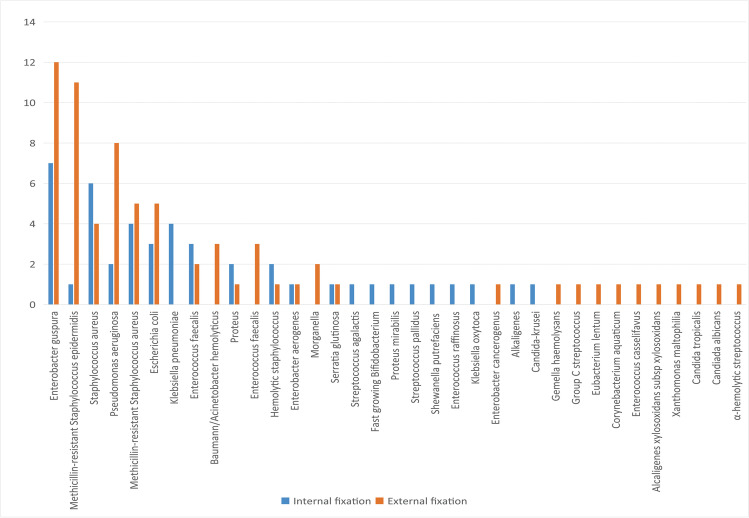
The distribution of pathogenic bacteria between two groups

A total of 85 people in both groups of patients had positive bacterial culture results, with a positive rate of 71.43%. Of these, 54 cases (21 in the internal fixation group and 33 in the external fixation group) had a monomicrobial infection. The most commonly isolated monomicrobial agent was *Enterobacter cloacae* in both groups (Fig. 2). Moreover, the results of positive bacterial cultures showed no significant difference between the two groups.

The pre-operative and post-operative haematological indexes were tested to assess the infection control. The post-operative CRP level in the internal fixation group was lower than that in the external fixation group, but others had no statistically significant difference (Table 4).

**Table 4:** Distribution of inflammatory markers

Parameters	Internal fixation (*n* = 56)	External fixation (*n* = 63)	*P* value
Before antibody treatment			
WBC (× 10^9^/L)	6.73 (5.27 ~ 10.18)	7.07 (5.54 ~ 9.83)	0.703
ESR (mm/h)	18.00 (7.25 ~ 59.00)	28.00 (33.00 ~ 53.00)	0.150
CRP (mg/L)	12.43 (1.94 ~ 31.47)	13.00 (4.63 ~ 36.50)	0.234
After antibody treatment			
WBC (× 10^9^/L)	6.47 (5.14 ~ 8.63)	6.21 (5.07 ~ 8.04)	0.682
ESR (mm/h)	18.00 (9.00 ~ 32.00)	23.00 (12.00 ~ 36.00)	0.204
CRP (mg/L)	3.16 (1.16 ~ 8.72)	6.30 (3.04 ~ 12.68)	0.009

## Discussion

The treatment of infected bone defects, especially large-size bone defects, remains a major challenge in the orthopaedic field. Traffic accidents and other high-energy factors lead to high fracture rates [22]. With the continuous improvement in medical technologies and wide application of fixation devices, most patients can receive surgical treatment as soon as possible. However, the subsequent post-operative infection has become an urgent issue in clinical practice. The incidence of post-infection can exceed 30% for the open fracture [23]. Treatment with internal fixation devices in the presence of infection is usually considered to be contraindicated because the internal fixation device is easily adhered by the residual bacteria, and once the biofilm is formed, it can prevent the antibiotic from contacting with bacteria and then lead to the recurrence of infection. External fixation is the mainstream stable method for treating infected bone defects at present [24], which can avoid the exposure of infected wounds to metal foreign bodies and facilitate repeated debridement. When a large defect exists, using telescoping external fixator following resection of the sequestrum can provide enough stability at the defect site. However, significant pain and restricted movement of the adjacent joints due to prolonged external fixation still exist. Studies have also shown that the long-term wearing of external fixation adversely affects the mental health and the quality of life of patients from many aspects [25, 26].

We used antibiotic bone cement–coated implants to better control the infection during the first-stage treatment of patients with infected bone defects compared with patients using external fixation. No significant difference was reported in the baseline data of the two groups of patients. In our study, the recurrence rate of infection after primary debridement was 7.14% in the internal fixation group and was 12.70% in the external fixation group; no statistically significant difference was observed. This might be because the sample size was inadequate. Hence, further large-scale prospective studies are needed to better explore the post-operative recovery of patients using internal fixation in the first stage of debridement.

We used the Paley criteria to evaluate the bone healing and limb function of the two groups of patients. The results showed that the limb function in the internal fixation group was better than that in the external fixation group. At the same time, we evaluated the post-operative SAS anxiety scale in the two groups of patients. The anxiety level in the two groups showed that the mental health of patients in the internal fixation group was better than that in the external fixation group. This was probably due to the following points: (1) Antibiotic bone cement formed a high concentration of antibiotics locally, which could effectively kill bacteria, prevent biofilm formation, and reduce the occurrence of infection [27]. (2) The patients in the internal fixation group could get out of bed early for functional exercise, and hence the joint stiffness and muscle atrophy caused by long-term external fixation were avoided; moreover, it had a favourable impact on the mental health of patients. (3) Early fixation provided favourable conditions for wound healing. Additionally, previous studies have shown that patients with chronic osteomyelitis often have marked osteopenia due to disuse [28]. For those patients, the use of antibiotic laden cement could fill dead spaces and avoid invasion of the affected area by poorly vascularized fibrous tissue and they help maintain normal anatomy (bony length and alignment, helping in the improvement of the trophism at the bone ends). Besides, the internal fixation device and bone cement allowed the gradual correction of osteoporosis of the affected limb in the functional recovery training after stage I surgery. (4) Internal fixation could provide more stability at the defect site compared with unilateral external fixation, although properly built external stabilization provided the possibility of weight bearing and exercises. But in clinical practice, we found that the internal fixation group was superior to the external fixation group in terms of weight-bearing capacity and mobility of the adjacent joints. The aforementioned results suggested that using internal fixation after primary debridement in patients with infected bone defects was feasible.

Although applying antibiotic-wrapped internal fixation for patients with infected bone defects after primary debridement has many advantages, different scholars have different views on the choice of internal fixation. Some scholars believe that intramedullary infection always occurs along the entire canal. Therefore, preventing infection through intramedullary contact is more effective. Our previous use of 3D-printed intramedullary nails wrapped with antibiotic bone cement for fixing infected bone defects after primary debridement has achieved good results [29]. Some scholars believe that if the intramedullary nail is used initially, it can affect the normal structure of the medullary cavity and thus interfere with the use of the intramedullary nail in the second-stage bone grafting operation [30].

Compared with intramedullary nails, locking plates have a wider range of applications, and it has little impact on the structure of the medullary cavity [9, 31]. In our study, 11 patients used intramedullary nailing and plate for internal fixation, 13 patients used plate alone, and 32 patients used intramedullary nail alone. It should be emphasized that on most occasions, we chose K-wire as the implant. The cement-wrapped inner K-wire core, which cannot provide sufficient stability, is usually combined with a low profile steel plate covered with bone cement on the outside, which not only ensures the occupation of the intramedullary nail but also improves the stability.

Since PMMA bone cement can generate a lot of heat energy during solidification, antibiotics with good thermal stability need to be added. Commonly used antibiotics in clinical practice include gentamicin, vancomycin, and tobramycin. No uniform standard exists for the dosage of antibiotics added to bone cement. Studies have shown that the amount of antibiotics added to each 40 g of PMMA bone cement should not exceed 8 g; otherwise, the mechanical strength of the bone cement is affected [32]. Antibiotics can act synergistically for better infection control [33]. Studies have shown that gentamicin is an ideal antibiotic for inclusion in PMMA because of its broad antimicrobial spectrum (Gram-positive and Gram-negative), low drug resistance rate, low protein binding rate, high water solubility, and thermal stability [34]. Therefore, most of the patients in our study used gentamicin bone cement, and vancomycin (for Gram-positive bacilli) or imipenem (for Gram-negative bacilli) was added based on the drug susceptibility and bacterial culture results of the patients for synergistic sterilization. According to our experience, bone cement has ideal mechanical strength when the dosage is no more than 4 g.

It is worth noting that the antibiotic carrier PMMA bone cement currently used clinically has the following shortcomings [35]: it is not absorbable, it requires a second surgery for removal, and the rate of antibiotic release is relatively fast, which cannot be sustained for a long time. Still, when antibiotic-laden cement is used, the antibiotic effect wears off over time, which can lead to resistance in the organism; some antibiotics are left in the bone cement that cannot be released, and heat is generated during the formation of bone cement. Since the stability of most is affected, they cannot be used in bone cement. Moreover, the drug release curve of antibiotic bone cement cannot be accurately predicted. In view of these shortcomings, an increasing number of researchers have turned their attention to the development of absorbable antibiotic sustained-release materials [36], exploring the combined use of biocompatible materials such as hydroxyapatite, calcium sulfate, calcium phosphate, and so on [37, 38]. A biodegradable antibiotic carrier with an ideal drug release rate is considered an effective strategy for the infection control of bone defects. In a retrospective study [39], 51 patients with chronic lower extremity osteomyelitis were divided into two groups. Vancomycin-loaded calcium sulfate and PMMA were combined to control the infection among patients with osteomyelitis in the experimental group, while only antibiotic-loaded PMMA was used in the control group. The result showed that the combined treatment in the experimental group achieved a synergistic effect in infection control.

The currently recognized fixation method at one-stage debridement of infectious bone defects is still an external fixator. However, many scholars advocate one-stage internal fixation after debridement; therefore, controversy exists. We conducted a retrospective case–control analysis, hoping to provide a basis for decision-making in this clinical controversy. Of course, this study also had shortcomings. It was a retrospective analysis conducted in a single centre. Hence, the sample size was not large enough, and the findings lacked the support of large-scale prospective studies.

## Conclusions

Based on the results of the study, we believed that the antibiotic bone cement–coated implant was a feasible fixation method for the primary treatment of infectious bone defects. It could provide better limb stability and patient compliance and reduce the psychological burden of patients compared with the external fixation, thereby improving the patient’s quality of life without increasing the rate of infection recurrence.

## Data Availability

The data used in this article are available from the corresponding author (Y.F.Z.) on reasonable request.
